# Integrated Transcriptomic and Proteomic Analyses Reveal the Role of NprR in *Bacillus anthracis* Extracellular Protease Expression Regulation and Oxidative Stress Responses

**DOI:** 10.3389/fmicb.2020.590851

**Published:** 2020-12-09

**Authors:** Yanchun Wang, Na Jiang, Bowen Wang, Haoxia Tao, Xin Zhang, Qing Guan, Chunjie Liu

**Affiliations:** ^1^State Key Laboratory of Pathogen and Biosecurity, Beijing Institute of Biotechnology, Beijing, China; ^2^Beijing Fisheries Research Institute, Beijing, China

**Keywords:** neutral protease regulator, extracellular proteases, *Bacillus anthracis*, transcriptomic, proteomic, oxidative stress responses

## Abstract

NprR is a protein of *Bacillus anthracis* that exhibits moonlighting functions as either a phosphatase or a neutral protease regulator that belongs to the RNPP family. We previously observed that the extracellular protease activity of an *nprR* deletion mutant significantly decreased within *in vitro* cultures. To identify the genes within the regulatory network of *nprR* that contribute to its protease activity, integrated transcriptomic and proteomic analyses were conducted here by comparing the *nprR* deletion mutant and parent strains. A total of 366 differentially expressed genes (DEGs) between the strains were observed via RNA-seq analysis. In addition, label-free LC-MS/MS analysis revealed 503 differentially expressed proteins (DEPs) within the intracellular protein fraction and 213 extracellular DEPs with significant expressional differences between the strains. The majority of DEGs and DEPs were involved in environmental information processing and metabolism. Integrated transcriptomic and proteomic analyses indicated that oxidation-reduction-related GO terms for intracellular DEPs and endopeptidase-related GO terms for extracellular DEPs were significantly enriched in the mutant strain. Notably, many genes involved in protease activity were largely downregulated in the *nprR* deletion mutant cultures. Moreover, western blot analysis revealed that the major extracellular neutral protease Npr599 was barely expressed in the *nprR* deletion mutant strain. The mutant also exhibited impaired degradation of protective antigen, which is a major *B. anthracis* toxin component, thereby resulting in higher protein yields. Concomitantly, another global transcriptional regulator, SpxA1, was also dramatically downregulated in the *nprR* deletion mutant, resulting in higher sensitivity to oxidative and disulfide stress. These data consequently indicate that NprR is a transcriptional regulator that controls genes whose products function as extracellular proteases and also is involved in oxidative stress responses. This study thus contributes to a more comprehensive understanding of the biological function of NprR, and especially in the middle growth stages of *B. anthracis*.

## Introduction

Extracellular proteases are secreted bacterial virulence factors that exhibit multiple roles in which they can attack host cells or tissues either directly or indirectly (Frees et al., 2013; Marshall et al., 2017). These proteases generally promote the breakdown of host tissues in order to promote bacterial dissemination into hosts and the colonization of deeper tissue layers or organs (Miyoshi and Shinoda, 2000; Vieira and Nascimento, 2016). Npr599 and InhA are the most important extracellular proteases for *Bacillus anthracis* virulence and enhance dissemination in addition to other activities (Chung et al., 2006, 2011). Npr599 and InhA first directly cleave extracellular matrix (ECM) components in order to degrade host tissues and increase barrier permeability. In addition, Npr599 and InhA activate syndecan-1 shedding by stimulating the target cell shedding mechanisms and accelerating the process through direct cleavage of the syndecan-1 ectodomain (Chung et al., 2006; Popova et al., 2006). Moreover, Npr599 and InhA can activate the fibrinolytic system via the human pro-urokinase plasminogen activator (pro-uPA) activation pathway or by cleaving the thrombin-activatable fibrinolysis inhibitor (TAFI), which has been observed in anthrax infections (Chung et al., 2011). Concomitantly, InhA1 can also directly activate prothrombin and factor X, resulting in blood coagulation (Kastrup et al., 2008). Perhaps more importantly, InhA can increase blood-brain barrier permeability and contribute to cerebral hemorrhages and is thus a critical virulence factor (Mukherjee et al., 2011). However, the expression and regulation of the *npr599* and *inhA* genes are uncharacterized.

NprR is a “moonlighting protein” of *Bacillus cereus* bacteria that functions as either a phosphatase or a neutral protease regulator and belongs to the RRNPP family that includes Rap, Rgg, NprR, PlcR, and PrgX (Rocha-Estrada et al., 2010; Rocha et al., 2012; Rice et al., 2016; Neiditch et al., 2017). NprR can modulate diverse activities by forming different polymeric structures. In general, mature NprX binds NprR and promotes the formation of a tetrameric structure of NprR that binds DNA and alters transcriptional activation of genes (Perchat et al., 2011; Zouhir et al., 2013). In the absence of NprX, NprR forms dimers and does not bind DNA, instead functioning as a phosphatase of Spo0F (Cabrera et al., 2016; Perchat et al., 2016). Furthermore, NprR is a component of NprR/NprX quorum sensing (QS) cell-cell communication that is controlled by the expression of the important neutral protease gene *nprA* (*npr599*) and a suite of other genes (Dubois et al., 2012; Perchat et al., 2016; Wu et al., 2017). When *B. anthraci*s Sterne colonies were incubated at 30°C for six or more days on LB agar plates, many papillation mutants were observed, and very few of them exhibited extracellular protease activities. The *nprR* genes in most of these mutants exhibited different mutational variations including point mutations, insertions, and deletions of various lengths. These mutational NprR could not act as neutral protease regulators, which strongly indicates that the mutations lead to the loss of the extracellular protease activities in *B. anthracis* (Yang et al., 2011). Moreover, *B. anthracis* V770-NP1-R (ATCC 14185), which has been used for efficient production of PA subunit vaccines, carries one nonsense mutation within the *nprR* gene (locus GBAA0597) that affects the expression of the neutral protease Npr599 (Cohen-Gihon et al., 2014).

Previously, we constructed the *nprR* gene deletion strain *B. anthracis* A16RΔ*nprR* (Wang et al., 2018). To further characterize the NprR regulatory system, comparative strand-specific RNA-seq and label-free LC-MS/MS were used in this study to identify genes that may be associated with the NprR regulons. Transcriptomic and proteomic analyses revealed that NprR is a critical regulator for bacterial protease expression. In addition, we observed for the first time that the NprR of *B. anthracis* is involved in stress response systems. This work contributes to our comprehensive understanding of the molecular mechanism underlying the activity and function of the neutral protease regulator NprR during bacterial growth.

## Materials and Methods

### Strains and Growth Conditions

The bacterial strains used in this study are shown in Table 1. *B. anthracis* A16RΔ*nprR* was previously described (Wang et al., 2018), while all other strains were constructed for this study and are described in further detail below. *Escherichia coli* DH5α was used for cloning experiments, and the plasmids used for electroporation of *B. anthracis* were prepared from *E. coli* SCS110. All strains were grown at 37°C with shaking at 220 rpm in Luria broth (LB). Antibiotics (Merck, Germany) were added to the media at appropriate final concentrations of erythromycin (400 μg/ml for *E. coli*, 5 μg/ml for *B. anthracis*), spectinomycin (50 μg/ml for *E. coli*, 300 μg/ml for *B. anthracis*), kanamycin (30 μg/ml for *B. anthracis*), and ampicillin (100 μg/ml, only for *E. coli*).

**TABLE 1:** Plasmids and strains used in this study.

Plasmids and strains	Relevant characteristics	Source
Plasmid		
pUC-PmtlA	pUC57 with PmtlA promoter	This lab
pBE2	Amp^*R*^, Kan^*R*^, *B. anthracis*–*E. coli* shuttle expression vector	This lab
pBE-nprR	pBE2 containing *nprR*	This work
pBE-spxA1	pBE2 containing *spxA1*	This work
*B. anthracis*		
A16R	pXO1^+^pXO2^–^, China vaccine strain	This lab
A16RΔ*nprR* (nprR)	A16R excision beginning 360 bp fragment of *nprR*	Wang et al., 2018
A16RΔ*nprR*(pBE-nprR) (c-nprR)	A16RΔnprR expressing *nprR* in plasmid pBE2	This work
A16RΔ*nprR*(pBE-spxA1) (c-spxA1)	A16RΔnprR expressing *spxA1* in plasmid pBE2	This work
A16RΔ*npr599* (npr599)	A16R deletion *npr599* gene	This lab
*E. coli* strains		
DH5α	cloning strain	CWBIO, China
SCS110	*dam*-/*dcm*- strain used to produce unmethylated plasmid	TransGen, China

### RNA Purification, Library Construction, and Sequencing

Three independent cultures from wild-type *B. anthracis* A16R and A16RΔ*nprR* were grown in LB over 12 h for transcriptomic experiments. Total RNA was isolated from cells using the TRIzol reagent (Invitrogen), followed by the treatment of extracts with RNase-free DNase I (Qiagen, Germany). rRNA was removed using a Ribo-Zero Magnetic kit (Epicentre, United States), and enriched mRNA was used to generate an RNA-Seq library using a TruSeq^TM^ Stranded Total RNA Library Prep Kit (Illumina, United States). The library was sequenced on an Illumina HiSeq 4000 platform at Majorbio (Shanghai, China), and the data were analyzed on the online Majorbio Cloud Platform^1^.

Clean sequence reads were mapped to the *B. anthracis* Sterne chromosome and the pXO1 plasmid using Bowtie. mRNA expression levels and transcripts per million (TPM) reads were calculated for each gene using RSEM (RNA-Seq by Expectation-Maximization). The TPM distribution density and correlation between the two groups were also analyzed to validate the reliability of the sequencing data.

Differentially expressed genes (DEGs) between A16R and A16RΔ*nprR* cultures were identified using DEseq2 with a Benjamini-Hochberg false discovery rate (FDR) used to determine the threshold *p* value for assigning statistical significance after multiple tests. A fold change value ≥2 and FDR ≤ 0.05 were used as thresholds for identifying significant differential expression.

### Functional Annotation of DEGs

Differentially expressed genes were subjected to Gene Ontology (GO) and KEGG enrichment analyses based on GO and KEGG pathway analysis using the Goatools and KOBAS software programs, respectively. The statistical significance of pathway enrichments was identified using Fisher’s exact test. *P* values from the GO and KEGG enrichment analyses were also corrected using multi-testing correction methods including Bonferroni, Holm, Sidak, and false discovery rate corrections. GO and KEGG pathways were considered significantly enriched when corrected *p* values were ≤0.05. All data were analyzed on the Majorbio Cloud Platform^2^.

### Protein Extraction and Label-Free LC-MS/MS Analysis

Three independent cultures from wild-type *B. anthracis* A16R and A16RΔ*nprR* were grown in 400 mL of LB over 14 h for proteomic analyses. The cell pellets and supernatant fractions of cultures were then separated by centrifugation at 17,000 *g* for 20 min at 4°C. To investigate extracellular proteins, the supernatant fraction was filtered using a 0.44-μm syringe filter, and the filtered growth medium was concentrated using ultrafiltration tubes (3 kDa MWCO, PALL). To investigate intracellular proteins, cell pellets were washed several times with PBS buffer and then resuspended in a urea-based extraction buffer (8.0 M urea with a protease inhibitor cocktail) and sonicated for 10 min following centrifugation at 12,000 *g* for 30 min at 4°C. Protein concentrations were estimated using a BCA Protein Assay Kit (Thermo Fisher Scientific, United States), and protein quality was assessed by sodium dodecyl sulfate (SDS)-polyacrylamide gel electrophoresis (PAGE). A 100 μg/vial protein sample was reduced and alkylated with 100 mM triethylammonium bicarbonate (TEAB) buffer along with 100 mM tris (2-carboxyethyl) phosphine (TCEP) and 40 mM iodoacetamide (IAM) by incubating for 40 min at room temperature in the dark. Six volumes of cold acetone were then added (cold acetone: sample v/v = 6:1), and proteins were precipitated for 4 h at −20°C. After centrifugation at 4°C for 3 min, the resultant precipitates were dissolved in 100 μL of 100 mM TEAB buffer.

Dissolved proteins were digested with sequencing grade trypsin (Promega) at an enzyme: substrate ratio of 1:50 (w/w) overnight at 37°C. After digestion, the peptides were vacuum-dried and dissolved in 0.1% trifluoroacetic acid (TFA), followed by desalting with an HLB SPE column (AISIMO) and drying by vacuum centrifugation. The concentrations of desalted peptides were then measured using a Pierce Quantitative Colorimetric Peptide Assay (Thermo Fisher Scientific) and dissolved in MS loading buffer (2% acetonitrile and 0.1% formic acid) to a final concentration of 0.25 μg/μL.

Peptides were separated with an acetonitrile gradient (2–80% over 120 min) in 0.1% formic acid using a reversed-phase analytical column (C18 column; Thermo Fisher Scientific), with a constant flow rate of 300 nl/min on an EASY-nLC 1200 UPLC system (Thermo Fisher Scientific, United States). Eluted peptides were analyzed on a Q Exactive HF-X mass spectrometer (Thermo Fisher Scientific, United States) operated in data-dependent acquisition (DDA) mode. Full-scan MS spectra were acquired between 350 and 1,300 m/z, with the 20 most intense signals per cycle fragmented. The resolutions of the MS and MS/MS scans were 60,000 and 15,000, respectively, and the dynamic exclusion was set to 18 s. Each sample was analyzed in triplicate.

The peptides were identified from the raw data using the Proteome Discoverer v.2.2 software program (Thermo Fisher Scientific). Software parameters were specified as follows: iodoacetamide for Cys alkylation, oxidation (M), and acetyl (protein N-terminus) for dynamic modification, carbamidomethyl for static modification, trypsin as the enzyme name, a maximum of two missed cleavage sites, 20 ppm for mass tolerance, and 1% false discovery rate. All data were analyzed on the Majorbio Cloud Platform (see text footnote 2). The mass spectrometry proteomics data have been deposited in the ProteomeXchange Consortium^3^ via the iProX partner repository with the dataset identifier PXD020800 (Ma et al., 2019).

### Construction of *nprR* and *spxA1* Complemented Strains

The genomic DNA of *B. anthracis* A16R was used as a template in order to amplify an *nprR* fragment with the promoter region using the nprRF and nprRR primers. The PmtlA promoter was amplified by mtlAF and mtlAR using pUC-PmtlA as template, while the spxA1 gene was amplified by spxA1F and spxA1R from A16R genomic DNA. All the sequences of the primers are listed in Table 2. The fragments were then cloned into the shuttle plasmid pBE2 to construct the expression plasmids for NprR and SpxA1. The expression plasmids were transformed into *B. anthracis* A16RΔ*nprR* as described previously (Wang et al., 2011).

**TABLE 2:** Primers used in this study.

Name	Sequence (5′→3′)	Purpose
nprRF	ACATGCATGCACAACTTCACCTTCTTGCATAC	PCR of *nprR* cassette
nprRR	CGGAATTCTTATTCCTCCTTATCATTC	
mtlAF	ACATGCATGCTTTATTTTTAAAAAATTGTC	PCR of PmtlA promoter
mtlAR	TTTGGTCTCTTTATATATTTCC	
spxA1F	TTTGGTCTCTATAATATGGTAACATTATATAGTTC	PCR of *spxA1* gene
spxA1R	CGGAATTCGAAGGTTTTCATATTATTATAA	
q-spxA1F	TGTACGTCTTGTAGAAAGGC	qPCR of spxA1 gene
q-spxA1R	AATAATCTCATCCGTTCCGC	
fusAF	TTGGTATCATGGCTCACATC	qPCR of fusA gene
fusAR	GCAGCAGAAGTAATTGTGAT	

### Phenotypic and Protein Expression Analysis

Extracellular protease activity phenotypes of the strains were investigated on milk plates, as previously described (Yang et al., 2011; Pineda-Castellanos et al., 2015). Briefly, fresh colonies grown on LB agar were used to inoculate an overnight LB culture (37°C, 220 rpm). The cultures (5 μl) were spotted onto milk plates and then incubated at 37°C. A distinct halo around the colonies was then identified at different time points as a phenotype.

To analyze the expression of secreted proteins like Npr599, InhA1, and PA, the *B. anthracis* strains were grown at 37°C in LB for 14 h, and secreted proteins were analyzed by Western blot. The supernatant fraction was collected from the bacterial cultures by centrifugation at 8,000 *g* for 10 min at 4°C, followed by filtering with a 0.45 μm syringe filter. The conditioned growth medium was concentrated 20-fold using Amicon Ultra-15 membranes (Millipore), and equivalent volumes (10 μL) of each sample were subjected to 12% SDS-PAGE. Separated proteins were transferred to an NC membrane (GE Healthcare, United States) using the Bio-Rad Trans-Blot system. The membranes were then incubated in blocking solutions containing 5% fat-free milk for 1 h and incubated with respective primary antibodies for 1.5 h at 37°C. After washing, the membranes were incubated with measurable IgG conjugated to HRP at a dilution of 1:5,000 (Jackson, United States). The membranes were treated with ECL reagents (Engreen Biosystem, Beijing, China) and visualized using a chemiluminescent imaging system (Tanon, China), according to the manufacturer’s instructions.

To analyze the expression of cytoplasmic proteins, cell pellets were resuspended using a 1/10 volume of PBS buffer and briefly sonicated followed by centrifugation at 16,000 *g* for 10 min at 4°C. The supernatant fractions were collected and analyzed by SDS-PAGE and western blot analysis, as described above. The expression of the ribosomal protein L6 was used as a loading control and was detected in samples from all strains (Wang et al., 2011).

### Quantitative Real-Time PCR

Quantitative real-time PCR analysis was performed to validate the RNA-seq data using RNA preparations that were generated as described above. The RNAs were subjected to cDNA synthesis using the HiScript Q RT SuperMix for qPCR (Vazyme, China). qPCR reactions were performed using a 2X ChamQ SYBR COLOR qPCR master mix (Vazyme, China) on a 7500 Fast Real-Time PCR System (Applied Biosystems, United States). All the sequences of the primers are listed in Table 2. Experimental duplicates were conducted for reactions using three independent cDNA preparations. Levels of expression of *spxA1* gene were normalized to a constitutively expressed gene (*fusA*). The efficiency of qPCR primers used was validated using thermal gradient and gel electrophoresis. The relative expression was calculated using the 2^–ΔΔ*Ct*^ method (Livak and Schmittgen, 2001).

### Stress Challenges

Fresh colonies grown on LB agar were used to inoculate overnight LB cultures at 37°C and 220 rpm to investigate the role of NprR in *B. anthracis* stress responses. Ten-fold serial dilutions of the cultures were spotted (5 μl) onto LB agar either supplemented or not supplemented with 200 μmol/L diamide, 0.44 mmol/L H_2_O_2_, or 0.0025% deoxycholate. Sensitivity on the LB agar plates to the supplemented chemicals was evaluated after 16 h of incubation at 37°C.

## Results

### Transcriptomic Analysis Following *nprR* Disruption

**FIGURE 1:**
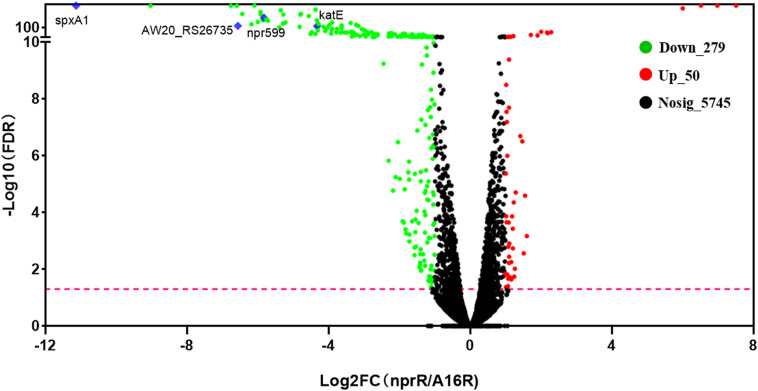
Volcano plots of differentially expressed genes (DEGs). Plots showing log2-fold change (FC) and –log_10_ FDR (corrected *p*-value) values of the DEGs when comparing the RNA-Seq profile of the *nprR* deletion mutant strain against that of the A16R strain. Green, red, and black points indicate downregulated, upregulated, and non-differentially expressed genes, respectively. The pink line represents FDR = 0.05. Some focused genes also were marked by blue diamond.

To evaluate the effect of *nprR* on transcriptomic profiles, RNA-Seq analysis was conducted using triplicate biological samples of the *nprR* mutant strain and A16R reference strain from early stationary phase cultures (∼ 12 h of growth). An average of 23.5 million clean reads was obtained per sample. All total mapping ratios and uniquely mapped ratios of clean reads to the *B. anthracis* Sterne strain genome were nearly 99%, indicating that sequencing was sufficient to cover all transcripts within the cells. Genome mapping revealed that 5,662 and 5,670 genes were expressed in the A16R and *nprR* mutant strain cells, respectively. A total of 5752 genes were detected and, among these, 5,580 were commonly expressed within the two groups. The distribution density of TPM values did not obviously differ between the two groups, and correlation analysis also illustrated a high degree of correlation of expression between the two samples (Supplementary Figure 1). Differentially expressed genes (DEGs) between the *nprR* mutant and A16R strains were identified that had at least 2-fold changes in expression (∣log_2_FC(NprR/A16R)∣ ≥ 1) and a corrected *p*-value (FDR) < 0.05 (Figure 1 and Supplementary Table 1). Gene expression comparisons indicated that 279 genes were significantly downregulated (green in Figure 1) and 50 were significantly upregulated (red in Figure 1) in the *nprR* deletion mutant relative to A16R cells. Only one gene on the plasmid pXO1 (AW20_RS00735) was downregulated in the mutant. The *npr599* gene, the direct target of NprR activation, was one of the most downregulated genes in the mutant (log_2_FC(NprR/A16R) = −5.81).

### GO and KEGG Enrichment Analysis of DEGs

**FIGURE 2:**
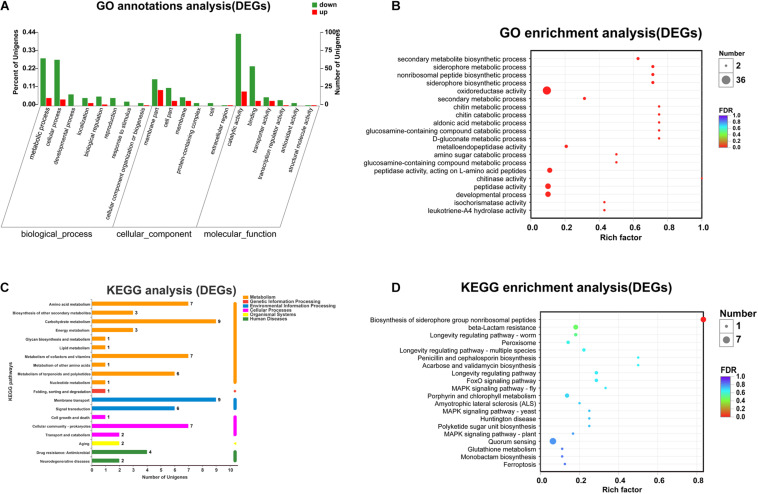
Functional annotation of the DEGs in the mutant and reference strains. **(A)** Top 20 terms of GO annotations. Red and green columns represent the number of upregulated and downregulated unigenes in GO terms, respectively. **(B)** GO functional enrichment analysis (The 20 most enriched GO terms). **(C)** KEGG pathway annotations. **(D)** KEGG pathway enrichment analysis. “Rich factor” means that the ratio of the DEGs number and the number of genes has been annotated in this term or pathway. The greater the Rich factor, the greater the degree of enrichment. The size of each point indicates the number of differentially expressed genes in that term (pathway).

To explore the functions of the DEGs and the pathways that they participate in, the DEGs were classified into GO categories, yielding three predominant classifications: biological process (BP), cellular component (CC), and molecular function (MF). Specifically, the DEGs were assigned to 24 functional groups by GO annotation (Figure 2A). GO functional enrichment analysis of DEGs was also conducted based on the annotation data. GO enrichment results of all DEGs were provided. A total of 24 GO terms were significantly enriched (corrected *p*-value, FDR < 0.05). In the top 20 GO terms, most of the terms were classified within the “biological process” and “molecular function” categories (Figure 2B). Among these, “secondary metabolite biosynthetic process,” “siderophore metabolic process,” “non-ribosomal peptide biosynthetic process,” “oxidoreductase activity,” “siderophore biosynthetic process,” and “secondary metabolic process” were the primary significantly enriched downregulated GO terms (FDR < 0.01), while the aldonic acid metabolic process and D-gluconate metabolic process were the primary significantly enriched upregulated GO terms (FDR < 0.01). KEGG pathway annotation and enrichment analysis were also used to evaluate significantly enriched DEGs (Figures 2C,D). Among the 20 most represented pathways for these DEGs, “biosynthesis of siderophore group non-ribosomal peptides” (pathway ID: map01053) was the only one that was significantly downregulated in *nprR* mutant strains (FDR = 0.0001).

### Proteomic Analysis and Identification of Differentially Expressed Proteins (DEPs)

**FIGURE 3:**
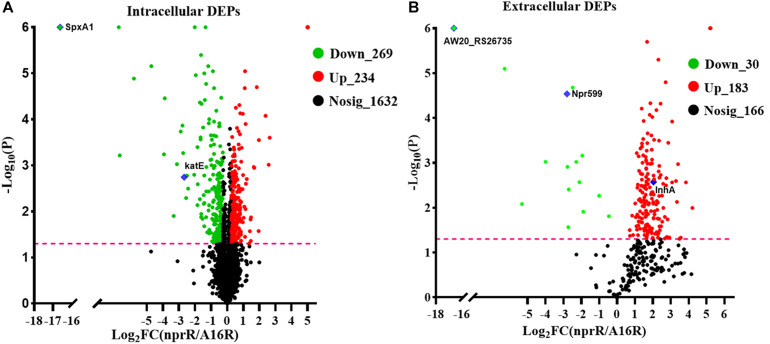
Volcano plots of differentially expressed proteins (DEPs) in the *nprR* deletion mutant and A16R strain. The plots show log2-fold change (FC) and –log_10_ (*p*-value) comparisons of the DEPs from the *nprR* deletion mutant strain against the A16R strain. A FC > 1.2 was used to identify upregulated DEPs and a FC < 0.83 for downregulated DEPs. **(A)** intracellular protein fraction. **(B)** Extracellular protein fraction. Green, red, and black points represent downregulated, upregulated, and non-differentially regulated proteins, respectively. The pink line represents FDR = 0.05. Some focused proteins are marked with blue diamonds.

To further understand the effects of nprR gene deletion on *B. anthracis* physiology, label-free LC-MS/MS analysis was used to evaluate the expression of intra- and extra-cellular proteins in the *B. anthracis* A16R and the *nprR* deletion mutant strains. DEPs were those exhibiting *P* < 0.05 and fold change (FC) >1.20 or <0.87 between groups. A total of 2,135 intracellular proteins were quantified, of which 503 were identified as DEPs, including 269 downregulated and 234 upregulated proteins in *nprR* deletion mutant strains (Figure 3A and Supplementary Table 2). Likewise, 379 extracellular proteins were identified, with 213 being DEPs and 30 downregulated, while 183 were upregulated (Figure 3B and Supplementary Table 3).

**FIGURE 4:**
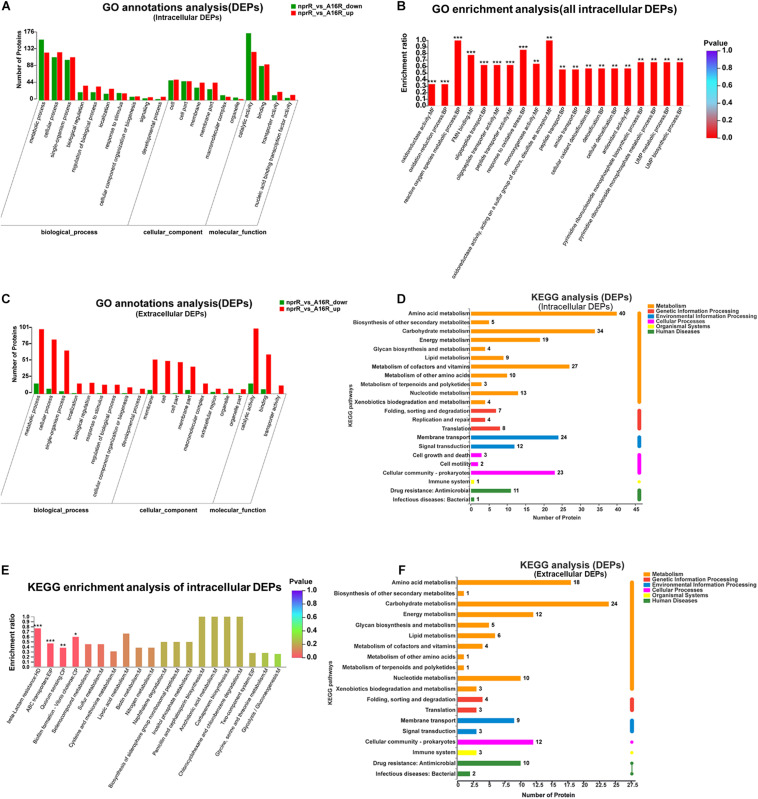
Functional classification of DEPs. **(A)** GO annotation of intracellular DEPs. **(B)** GO functional enrichment analysis of intracellular DEPs. **(C)** GO annotation of extracellular DEPs. **(D)** KEGG annotation of intracellular DEPs. **(E)** KEGG pathway enrichment analysis of intracellular DEPs. **(F)** KEGG pathway annotations of extracellular DEPs. *P < 0.05, **P < 0.01, ***P < 0.001.

GO annotation revealed that the 503 intracellular DEPs were assigned to 35 upregulated GO terms and 30 downregulated. The 20 most abundant GO terms (based on protein abundances) are shown in Figure 4A. GO functional enrichment analysis of the DEGs was also conducted using the annotation data (Figure 4B and Supplementary Table 4). The 20 most significantly enriched GO terms were primarily associated with the BP and MF categories and included “oxidoreductase activity,” “oxidation-reduction process,” “reactive oxygen species metabolic process,” “FMN binding,” “oligopeptide transport,” “oligopeptide transporter activity,” and “response to oxidative stress” (*p* < 0.001). In addition, GO term analysis of the 279 extracellular DEPs resulted in an annotation of 39 upregulated and 21 downregulated GO terms, and the 20 most abundant GO terms are shown in Figure 4C. However, only two of these were significantly differentially enriched and were associated with “protein binding” and “endopeptidase activity” (Supplementary Figure 2). In addition to the GO term analysis, KEGG pathway analysis was also conducted to assign the DEPs of the *nprR* deletion mutant and A16R strains to metabolic pathways. A total of 503 intracellular DEPs were assigned into 85 KEGG pathways, the majority of which were associated with metabolism (M) (Figure 4D). Four specific KEGG pathways comprising “beta-lactam resistance,” “ABC transporters,” “quorum sensing,” and “biofilm formation” were significantly enriched in the DEP profiles (FDR < 0.05) (Figure 4E). Extracellular DEPs were also assigned to 61 KEGG pathways, but none were significantly enriched between groups (Figure 4F).

### Integrated Transcriptomic and Proteomic Analysis

**FIGURE 5:**
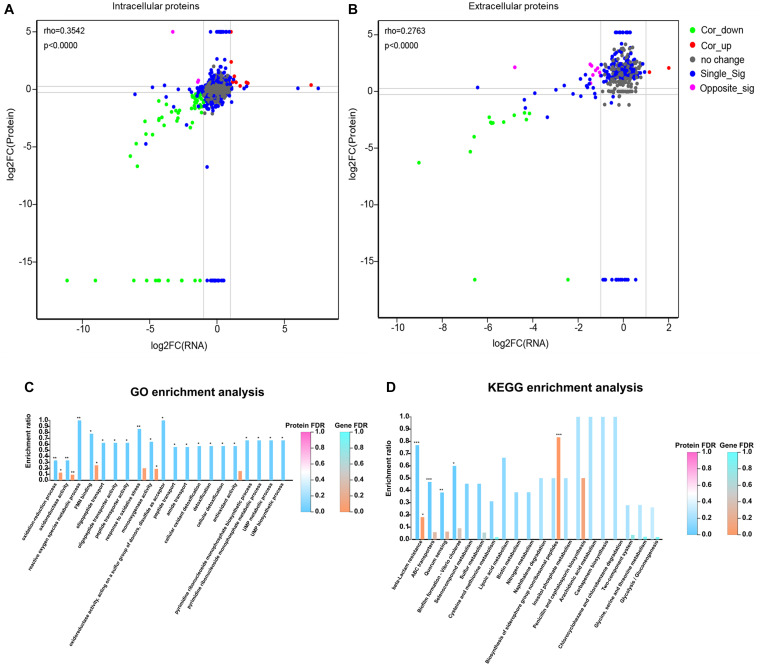
Integrated analysis of the RNA-seq and label-free MS data for comparisons of the mutant and reference strains. **(A,B)** Correlation analysis between the RNA-seq and MS data for intracellular and extracellular protein fractions, respectively. In the nine-quadrant diagram, green (Cor_down) represents downregulated genes and proteins, red (Cor_up) represents upregulated genes and proteins, gray (no change) represents genes and proteins with no significant difference, blue (Single_Sig) represents one of the genes and proteins with no significant difference, purple (Opposite_Sig) indicates that DEGs and DEPs show opposite upregulation and downregulation. **(C)** Integrated GO term enrichment analysis of DEGs and DEPs for the intracellular protein fraction. **(D)** Integrated KEGG pathway enrichment analysis of DEGs and DEPs for the intracellular protein fraction. *FDR < 0.05, **FDR < 0.01, ***FDR < 0.001.

To better understand the correlation between mRNA and protein expression profiles, an integrated analysis of the proteomic and transcriptomic data was conducted and the results are listed in Supplementary Tables 5, 6. Pearson correlation coefficient *rho* was used to assess the correlation between the proteomic and transcriptomic data. All of the quantified proteins, including 2,135 intracellular and 379 extracellular proteins corresponded to those inferred from the RNA-seq data. The distribution of the corresponding mRNA: protein ratios is shown in a scatterplot of the log2-transformed ratios. For intracellular proteins, the integration analyses data revealed that 57 genes (green plot) and their corresponding proteins were downregulated, while 10 (red plot) were upregulated (Figure 5A). Likewise, among extracellular proteins, there were 14 upregulated and two downregulated (Figure 5B) genes/proteins that had corresponding changes of expression. The details of the genes and proteins that had corresponding changes in expression are listed in Supplementary Tables 7, 8. As in many other studies (Haider and Pal, 2013), a poor positive correlation was observed between transcript and protein abundances in the mutant and A16R strains, with rho = 0.3542 for intracellular proteins and rho = 0.2763 for extracellular proteins (Figures 5A,B). Integrated analyses were subsequently conducted based on the DEPs and DEGs functional enrichment analysis in order to explore the correlations between the proteomes and transcriptomes from the perspective of protein function. Significantly enriched GO terms for intracellular proteins (FDR < 0.05) for both DEGs and DEPs are shown in Figure 5C. The GO terms “oxidoreductase activity,” “oxidation-reduction process,” “FMN binding,” and “monooxygenase activity” were significantly enriched in both the DEPs and DEGs profiles, while the KEGG pathway “beta-lactam resistance” was the only pathway significantly enriched in both the DEPs and DEGs profiles (Figure 5D). Regarding extracellular proteins, “endopeptidase activity” was the only GO term significantly enriched in both the extracellular DEGs and DEPs profiles (Supplementary Figure 3, FDR < 0.05), and no KEGG pathways were significantly enriched in the extracellular DEGs and DEPs profiles. This result was fully consistent with the priorities of neutral protease regulators.

### The Deletion of *nprR* in *B. anthracis* Decreases Proteolytic Enzyme Activity

**FIGURE 6:**
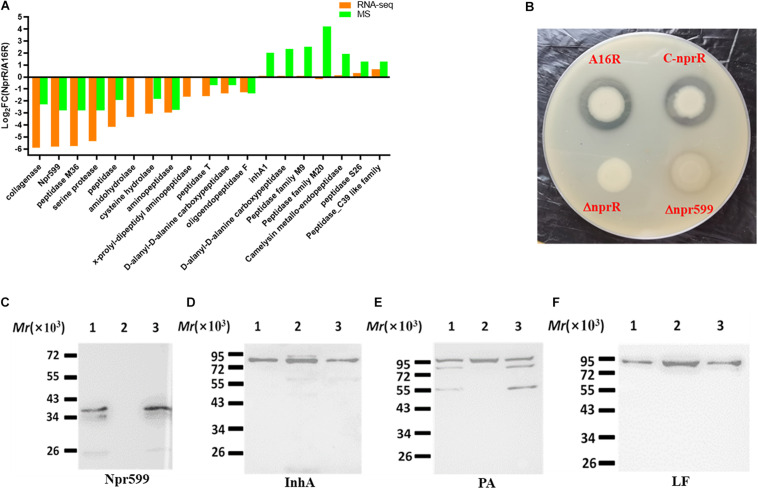
Analysis of protease-related phenotypic characteristics of the *nprR* deletion mutant. **(A)** Comparison of protease-related DEGs and DEPs in the *nprR* deletion mutant strain against the A16R strain. **(B)** Extracellular proteolytic enzyme activity assays for different strains. Strain colonies with considerable extracellular proteolytic enzyme activity are surrounded by halos. **(C–F)** Western blot analysis to evaluate the expression of important *B. anthracis* proteins including Npr599 **(C)**, InhA **(D)**, PA **(E)**, and LF **(F)**. Lanes are indicated as follows: 1, A16R; 2, the *nprR* deletion mutant of A16R, 3, the *nprR* recovered strain. All Western blot experiments were performed at least three times.

Some DEGs and DEPs were clearly associated with protease activity based on annotation information. To better understand the regulation of protease-associated protein expression by NprR, several downregulated genes involved in protease activity identified in the RNA-seq and MS analyses were further evaluated (Figure 6A). Numerous proteins (including Npr599 and a peptidase M4 family protein) that were associated with protease activity were downregulated in the *nprR* deletion mutant strain. Furthermore, the MS analysis indicated that many proteases (peptidases) were also upregulated in the *nprR* deletion mutant strain, but not in RNA-seq result (Figure 6A). In particular, the InhA protein, another important protease for *B. anthracis* infection, was upregulated in the *nprR* deletion mutant strain relative to the A16R strain. Consequently, the extracellular proteolytic activity of strains was evaluated using phenotypic tests on skim milk agar media. Extracellular enzymatic activity is expressed as a halo (i.e., a zone of hydrolysis) around colonies in this test. Based on halo size, the *nprR* deletion mutant strain exhibited a large decrease in extracellular proteolytic enzymatic activity compared to the original A16R strain (Figure 6B). In contrast, the *nprR* complemented strain exhibited similar extracellular proteolytic activity as the A16R strain, indicating that plasmid-expressed NprR could recover the extracellular proteolytic enzyme activity of *nprR* deletion mutant strains. *npr599* encodes the neutral protease Npr599, which is the most important gene regulated by NprR. However, the *npr599* deletion mutant strain had weak extracellular proteolytic activity compared to the *nprR* deletion mutant strain, but the latter had almost no extracellular enzymatic activity. Thus, Npr599 is not the only extracellular proteolytic enzyme regulated by NprR, and others are also downregulated by this important neutral protease regulator.

To further investigate the effects of extracellular proteolytic enzymes on the expression of other important proteins, the culture media of the *nprR* deletion mutant was used for further western blot analysis by recovering equal concentrations of protein samples from both the mutant and A16R strain cultures. The expression of the neutral protease Npr599 was almost undetectable in the *nprR* deletion mutant strain compared to the A16R strain. In addition, *nprR* complementation resulted in normal Npr599 expression relative to the A16R strain (Figure 6C). In contrast, the expression of another important extracellular proteolytic enzyme, InhA, was only slightly upregulated (Figure 6D). These results were mostly consistent with the RNA-seq [*npr599*, log_2_FC(NprR/A16R) = −5.82; *inhA1*, log_2_FC(NprR/A16R) = 0.06] and MS [Npr599, log_2_FC(NprR/A16R) = −2.8; InhA, log_2_FC(NprR/A16R) = 2.03] analyses. In addition, the degradation of protective antigen (PA) proteins was significantly reduced in the mutant strains, while complemented NprR expression in the *nprR* deletion mutant led to similar degradation as the A16R strain (Figure 6E). Concomitantly, the lethal factor (LF) expression level was also slightly upregulated (Figure 6F). These results generally coincided with those of the MS analysis [PA, log_2_FC(NprR/A16R) = 1.536; LF, log_2_FC(NprR/A16R = 1.458)].

### Deletion of NprR in *B. anthracis* Increases Oxidative Stress Susceptibility

**FIGURE 7:**
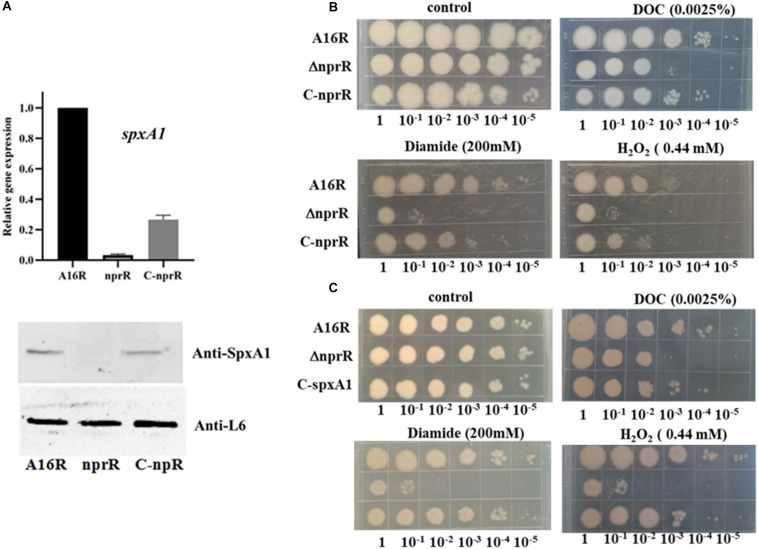
Oxidative stress susceptibility analysis of the *nprR* deletion mutant. **(A)** qPCR and western blot analysis of *spxA1* expression. **(B,C)** Strains were grown on LB media with or without 0.0025% DOC, 200 mmol/L diamide, or 0.44 mmol/L H_2_O_2_. Five microliters at the indicated dilutions were spotted onto LB agar (Materials and Methods). All experiments were performed at least three times.

The *spxA1* gene was significantly downregulated according to the RNA-seq and MS analyses [log_2_FC(NprR/A16R) = −11.13 and −16.61, respectively]. qPCR and western blot analyses were thus used to validate these results. The expression of SpxA1 was almost undetectable in the *nprR* deletion mutant strain compared to the A16R and *nprR* complemented strains (Figure 7A). Because cells lacking *spxA1* are sensitive to oxidative stress, an oxidative stress susceptibility test was applied to the *nprR* mutant strain using several oxidants in culture agar plates. The mutant strain was more sensitive to peroxide, diamide, and salt deoxycholate relative to the A16R strain (Figure 7B). Thus, deletion of *nprR* made the strain more sensitive to oxidative stress and disulfide formation. To explore whether the loss of stress resistance in the *nprR* mutant is due to the downregulation of *spxA1* gene directly, the *nprR* mutant complemented with an SpxA1 expression plasmid was constructed. Oxidative stress susceptibility testing showed that defects in peroxide, diamide, and salt deoxycholate resistance could be corrected by introducing *spxA1* in plasmid, suggesting that downregulation of SpxA1 protein might be the main reason that the *nprR* deletion mutant strain was more sensitive to oxidative stress (Figure 7C). RNA-seq and MS analyses also indicated that the expression levels of AW20_RS26735 encoding peroxidase [log_2_FC(NprR/A16R) = −6.563 or −16.61, respectively] and AW20_RS27305 encoding catalase [*katE*, log_2_FC(NprR/A16R) = −4.323 or −2.679, respectively] were significantly downregulated in the *nprR* deletion mutant strain compared to the A16R strain (Figures 1, 3).

## Discussion

NprR is an important regulatory protein of *B. anthracis*, with a primary function to regulate the expression of extracellular proteases like Npr599 in the early stages of bacterial growth (Rice et al., 2016). Here, we evaluated the role of this regulatory factor in the post-exponential growth phase. To explore the mechanism of NprR regulation of extracellular protease expression, an *nprR* deletion mutant was used to systematically investigate downstream genes regulated by NprR using RNA-seq and label-free MS approaches, with targeted validation by qPCR and western blot experiments. The analyses yielded clarification of NprR function during the middle stages of bacterial growth. Extracellular protease activity of the *nprR* deletion mutant was less than that of the *npr599* mutant. Thus, in addition to the downstream gene *npr599* that is regulated by NprR, the expression of other genes involved in extracellular protease activity was also likely regulated, which was supported by the RNA-seq and MS analyses. For example, the only GO term significantly enriched in both the DEGs and DEPs (*P* < 0.05) was “endopeptidase activity.” Nevertheless, exceptions to this activity were also observed. Specifically, InhA is another important extracellular protease, and its expression was significantly upregulated based on the MS analysis, although mRNA levels did not change in the RNA-seq analyses. We speculate that this difference may primarily arise because InhA is one of the targets of Npr599, so that when the expression of Npr599 was significantly downregulated, InhA expression was upregulated due to reduced enzyme digestion effects.

In our opinion, the protein degradation caused by Npr599 was the main reason for the decrease in the amounts of some extracellular protein, such as PA and LF, and the degradation made it possible for this mutant strain to develop an effective host for production of recombinant anthrax antigen proteins (Pomerantsev et al., 2011). In addition, owing to the downregulation of Npr599 expression in the *nprR* mutant strain, the degradation of many extracellular proteins was significantly reduced, but there were no readily visible differences in the transcriptional level of these genes (coded extracellular proteins) between mutant strain and parental strain A16R. This might explain why the positive correlation between transcript and protein abundances was poor, especially regarding extracellular proteins (Figures 5A,B).

We also observed that NprR is associated with the oxidative stress response of *B. anthracis*. RNA-seq analysis indicated that the expression level of the *spxA1* (AW20_RS08630) and *spxA2* (AW20_RS25945) genes significantly downregulated in the *nprR* deletion mutant strain. Both SpxA1 and SpxA2 are closely related to the oxidative stress responses of *B. anthracis*, and *spxA1* is essential for peroxide resistance and participates in disulfide stress tolerance (Barendt et al., 2013, 2016). SpxA1 is also involved in oxidative stress response of many gram-positive bacteria (Chen et al., 2012; Kajfasz et al., 2017; Nilsson et al., 2019; Cesinger et al., 2020). For example, Chen et al. (2012) reported that SpxA1 is a global regulator required to activate genes encoding catalase in *Listeria monocytogenes*, and Nilsson et al. (2019) also reported that SpxA1 is involved in the oxidative stress response in *Streptococcus mutans*. As far as our results are concerned, SpxA1 exhibited the greatest change in expression levels in the mutant strain compared to the control strain, according to the RNA-seq and MS analyses. So that we speculated that *spxA1* is a newfound target gene of the moonlight protein NprR. At the same time, our result also indicated that the defects of *nprR* mutant in peroxide, diamide and salt deoxycholate resistance could be corrected by introducing spxA1 in plasmids and the downregulation of SpxA1 protein might be the direct reason that the *nprR* deletion mutant strain was more sensitive to oxidative stress. However, it remains unclear whether NprR protein downregulated the expression of *spxA1* directly or indirectly. It is necessary to determine whether the signal polypeptide NprX plays an important role in this process. The biological mechanism underlying the omics data merits in-depth study in our future work.

Integrated transcriptomic and proteomic analyses also showed that most significantly enriched GO terms for intracellular proteins were associated with oxidative stress responses. We thus speculated that the mutant strain was more sensitive to oxidative and disulfide stress, and subsequent phenotypic experiments confirm this hypothesis. Specifically, the mutant strain was more sensitive to oxidants like hydrogen peroxide and deoxycholate. This sensitivity was reflected in the significantly inhibited growth of the mutant relative to the A16R and complemented strains when exposed to the same concentrations of hydrogen peroxide. The RNA-seq and MS analyses also suggested that the expression levels of peroxidase (AW20_RS26735) and catalase (katE, AW20_RS27305) were significantly downregulated in the mutant (Figure 1), thereby suggesting that degradation of hydrogen peroxide was impaired in the mutant strain. These results may explain why the mutant strain is more sensitive to hydrogen peroxide. Previous work showed that the analogous genes were also downregulated in *spxA1* deletion mutant and these mutant strains were more sensitive to hydrogen peroxide in *Streptococcus sanguinis* and *Listeria monocytogenes* than wild-type strains (Chen et al., 2012; Cesinger et al., 2020). The mechanism by which BprR protein regulates these genes remains unclear, and further work is still needed to provide an adequate explanation. The RNA-seq and MS analyses from this study will provide useful reference materials for future related oxidative stress responses research in *B. anthracis*.

NprR protein is a typical moonlight protein. It might also be involved in many other biological processes, not including regulation the expression of extracellular proteases. For instance, in the GO term and KEGG pathway analyses, several DEGs and DEPs involved secondary metabolic process, such as siderophore biosynthetic and metabolic process, were enriched (Figures 2, 4). Siderophore is a secondary metabolite secreted by microorganisms. It serves primarily to transport iron across cell membranes. According to published work, *B. anthracis* requires siderophore biosynthesis for growth in macrophages and for mouse virulence (Cendrowski et al., 2004). Normally, siderophore biosynthesis occurs via two pathways: the non-ribosomal peptide synthetase (NRPS) pathway and the NRPS-independent siderophore (NIS) synthetase pathway (Carroll and Moore, 2018). Coincidentally, several DEGs involving non-ribosomal peptide biosynthetic process were also enriched in GO term enrichment analyses. These results indicated that the NprR protein might also play an important role in the NRPS-dependent siderophore biosynthesis of *B. anthracis*. Siderophore synthesis can also be regulated by oxidative stress in some microorganisms, including *B. anthracis* (Tindale et al., 2000; Lee et al., 2011; Adler et al., 2014). We speculate that there may be a connection between the downregulation of genes related to NRPS-dependent siderophore biosynthesis and the genes related to oxidative stress responses in the *nprR* mutant strain of *B. anthracis* and the corresponding mechanism and explanation of these results will be explored in greater depth in our next work.

## Data Availability Statement

The datasets presented in this study can be found in online repositories. The names of the repository/repositories and accession number(s) can be found below: ProteomeXchange [accession: PXD020800].

## Author Contributions

YW and CL designed the research. YW, NJ, HT, BW, QG, and XZ performed all the experiments. YW, NJ, and CL analyzed the data. YW wrote the manuscript. All authors reviewed the final manuscript.

## Conflict of Interest

The authors declare that the research was conducted in the absence of any commercial or financial relationships that could be construed as a potential conflict of interest.

## Footnotes

1 www.majorbio.com

2 https://cloud.majorbio.com/

3 http://proteomecentral.proteomexchange.org

## Supplementary Material

Supplementary Figure 1 Summary of the RNA-seq analyses of the nprR deletion mutant and A16R strains. **(A)** Venn diagram, **(B)** distribution density analysis, and **(C)** correlation analysis showing the overlap in gene expression between the two strains.

Supplementary Figure 2 GO term enrichment analysis of the extracellular DEPs. MF, molecular function. ^∗^*P* < 0.05.

Supplementary Figure 3 Integrated GO term enrichment analysis of DEGs and DEPs for the extracellular protein fraction. ^∗^FDR < 0.05.

Supplementary Table 1 The detail of differentially expressed genes (DEGs) between the *nprR* mutant and A16R strains.

Supplementary Table 2 The detail of intracellular differentially expressed proteins (DEPs) between the *nprR* mutant and A16R strains.

Supplementary Table 3 The detail of extracellular differentially expressed proteins (DEPs) between the *nprR* mutant and A16R strains.

Supplementary Table 4 GO functional enrichment analysis data of the DEGs.

Supplementary Table 5 Integrated analysis data of DEGs and DEPs (intracellular proteins).

Supplementary Table 6 Integrated analysis data of DEGs and DEPs (extracellular proteins).

Supplementary Table 7 The details of the DEGs and intracellular DEPs that had corresponding changes.

Supplementary Table 8 The details of the DEGs and extracellular DEPs that had corresponding changes.
