# Supplementary material for: Integrated Transcriptomic and Proteomic Analyses Reveal the Role of NprR in Bacillus anthracis Extracellular Protease Expression Regulation and Oxidative Stress Responses
Source: Front Microbiol. 2020 Dec 9;11:590851. doi: 10.3389/fmicb.2020.590851 (PMC7756075; doi:10.3389/fmicb.2020.590851)
Supplement: Supplementary Figure 1 — Summary of the RNA-seq analyses of the nprR deletion mutant and A16R strains. (A) Venn diagram, (B) distribution density analysis, and (C) correlation analysis showing the overlap in gene expression between the two strains. [file Data_Sheet_1.docx]

**
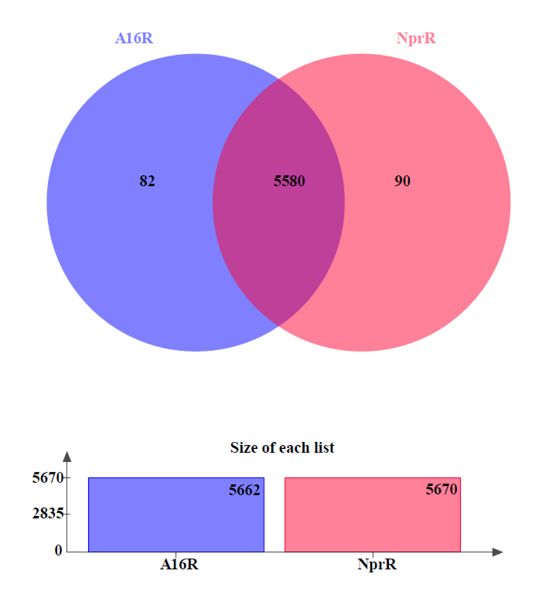
**

A

**
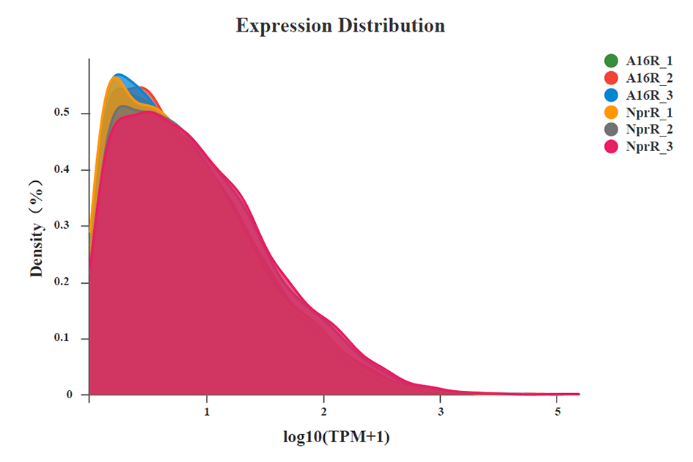
**

B

**
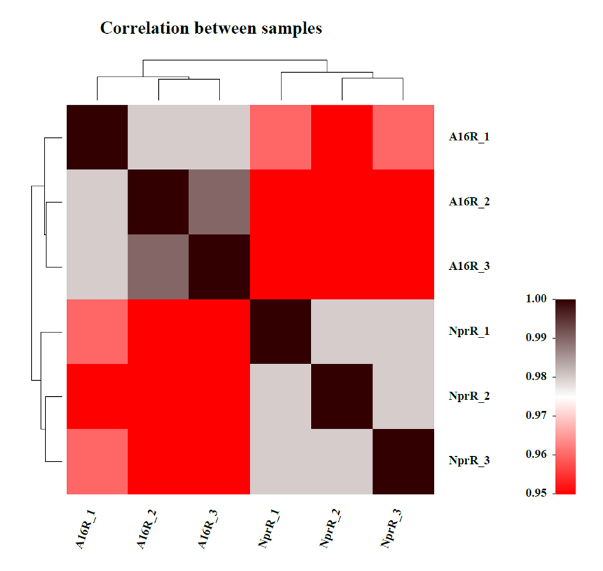
**

C

Figure S1: Summary of the RNA-seq analyses of the *nprR* deletion mutant and A16R strains. (A) Venn diagram, (B) distribution density analysis, and (C) correlation analysis showing the overlap in gene expression between the two strains.


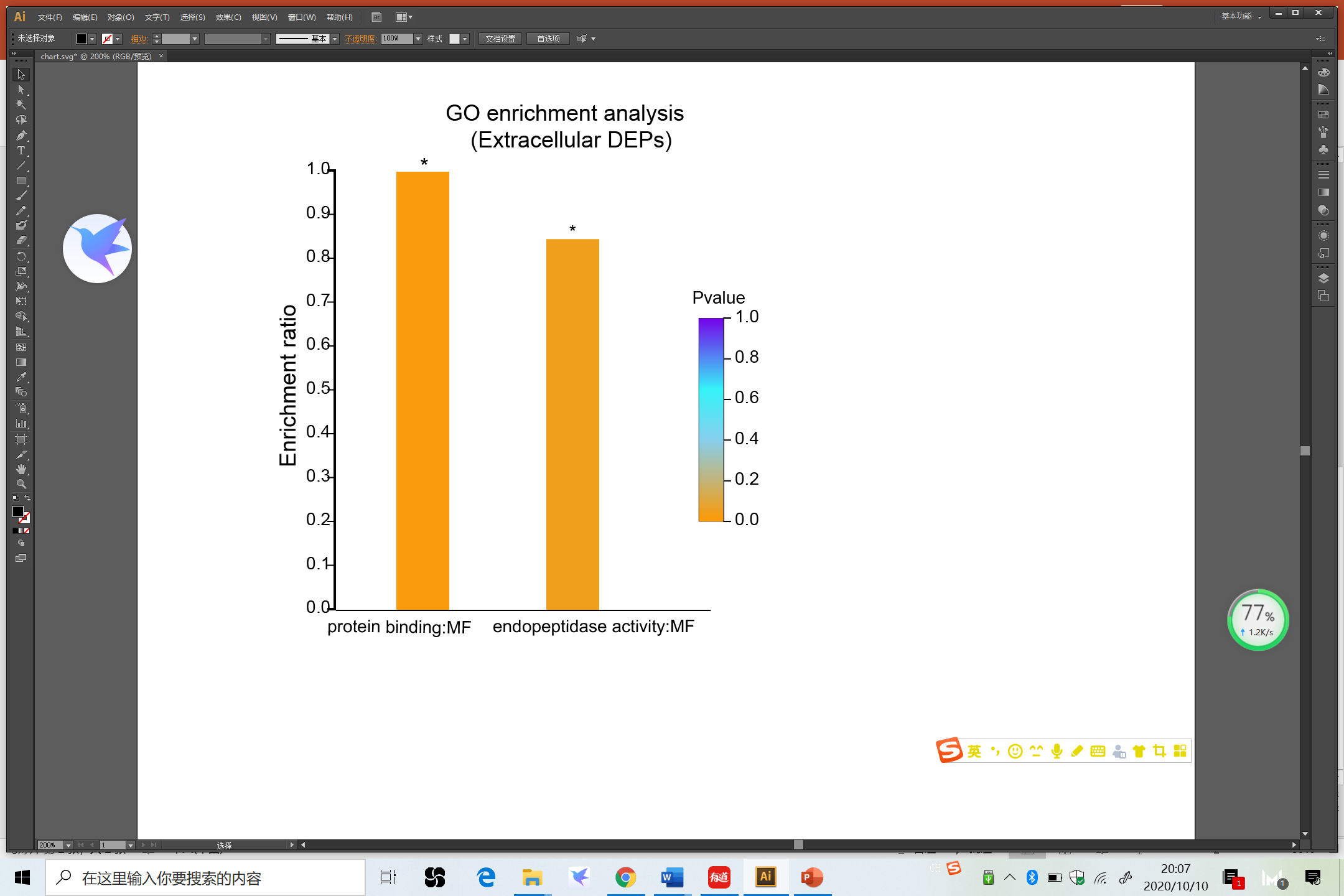


Figure S2. GO term enrichment analysis of the extracellular DEPs. MF, molecular function. * *P* < 0.05.


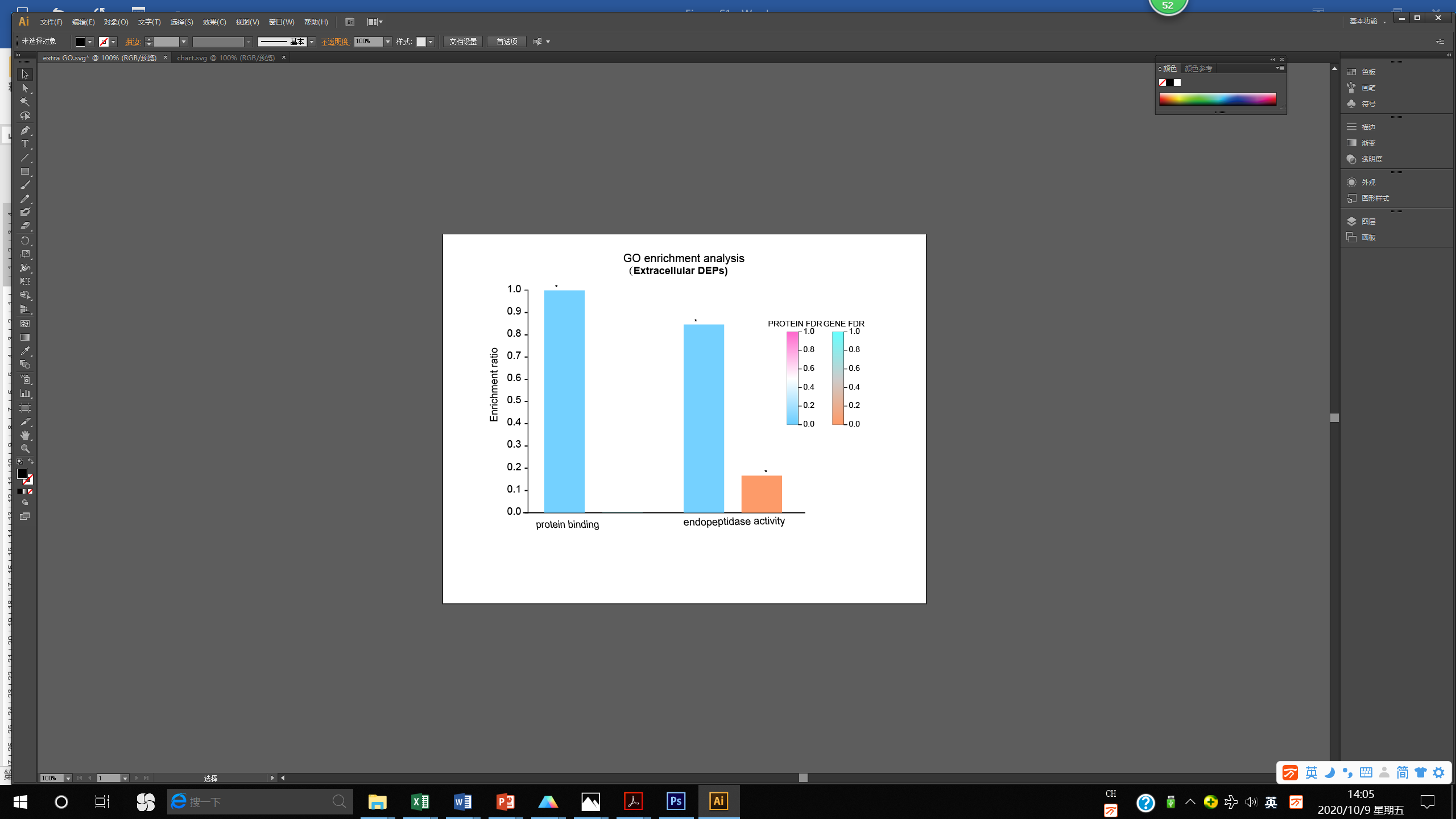


Figure S3. Integrated GO term enrichment analysis of DEGs and DEPs for the extracellular protein fraction. * FDR < 0.05
